# Preparation, Spectrochemical, and Computational Analysis of L-Carnosine (2-[(3-Aminopropanoyl)amino]-3-(1*H*-imidazol-5-yl)propanoic Acid) and Its Ruthenium (II) Coordination Complexes in Aqueous Solution

**DOI:** 10.3390/molecules161210269

**Published:** 2011-12-09

**Authors:** Michael Lee Branham, Parvesh Singh, Krishna Bisetty, Myalo Sabela, Thirumala Govender

**Affiliations:** 1 School of Pharmacy and Pharmacology, University of KwaZulu-Natal, Durban, 4001, South Africa; Email: govenderth@ukzn.ac.za (T.G.); 2 Department of Chemistry, Durban University of Technology, Durban, 4001, South Africa; Email: parveshdurban@gmail.com (P.S.); bisettyk@dut.ac.za (K.B.); myalosabela@dut.ac.za (M.S.)

**Keywords:** carnosine, ^13^C-NMR, ^1^H-NMR, ^15^N-NMR, metallopeptides, ruthenium complexation

## Abstract

This study reports the synthesis and characterization of novel ruthenium (II) complexes with the polydentate dipeptide, L-carnosine (2-[(3-aminopropanoyl)amino]-3-(1*H*-imidazol-5-yl)propanoic acid). Mixed-ligand complexes with the general composition [ML_p_(Cl)_q_(H_2_O)_r_]·xH_2_O (M = Ru(II); L = L-carnosine; p = 3 − q; r = 0–1; and x = 1–3) were prepared by refluxing aqueous solutions of the ligand with equimolar amounts of ruthenium chloride (black-alpha form) at 60 °C for 36 h. Physical properties of the complexes were characterized by elemental analysis, DSC/TGA, and cyclic voltammetry. The molecular structures of the complexes were elucidated using UV-Vis, ATR-IR, and heteronuclear NMR spectroscopy, then confirmed by density function theory (DFT) calculations at the B3LYP/LANL2DZ level. Two-dimensional NMR experiments (^1^H COSY, ^13^C gHMBC, and ^15^N gHMBC) were also conducted for the assignment of chemical shifts and calculation of relative coordination-induced shifts (RCIS) by the complex formed. According to our results, the most probable coordination geometries of ruthenium in these compounds involve nitrogen (N1) from the imidazole ring and an oxygen atom from the carboxylic acid group of the ligand as donor atoms. Additional thermogravimetric and electrochemical data suggest that while the tetrahedral-monomer or octahedral-dimer are both possible structures of the formed complexes, the metal in either structure occurs in the (2+) oxidation state. Resulting RCIS values indicate that the amide-carbonyl, and the amino-terminus of the dipeptide are not involved in chelation and these observations correlate well with theoretical shift predictions by DFT.

## 1. Introduction

L-Carnosine (2-[(3-aminopropanoyl)amino]-3-(1*H*-imidazol-5-yl)propanoic acid) [1] is a naturally occurring dipeptide of considerable biological and pharmacological importance. It is composed exclusively of covalently bonded alanine and histidine residues, and is found in the brain, heart, skin, muscle and gastrointestinal tissues. Although the precise biological role of carnosine is not completely understood, many studies indicate that it has extensive antioxidant potential [2] and is capable of decreasing intracellular reactive oxygen species (ROS) levels. Carnosine may also act as a neurotransmitter, and recent studies [2,3,4] suggest that it possesses remarkable wound healing properties. The physiological behavior and bioactivities of carnosine depend on its complexation of metal cations. Carnosine is a polydentate ligand with five potential metal-coordinating sites, *i.e.*, the two imidazole nitrogens, one carboxylate group, a peptide linkage, and a terminal amino group. Both tetrahedral and octahedral types of complexes can be formed, but their exact configuration depends on size of the metal cation, ligand-to-metal ratios, and the ionic strength of the supporting solution [5]. Carnosine complexation with numerous divalent cations other than Ru^2+^ (e.g., Cu^2+^, Zn^2+^, Co^2+^, Ni^2+^, Mn^2+^, Cd^2+^, Mg^2+^, and Ca^2+^) have been reviewed [5,6,7]; crystal structures of L-carnosine [8] and its Cu^2+^ complex [9] have also been elucidated.

Organoruthenium complexes are currently being rigorously investigated because of their application as heterogenous catalysis [10] and several recent reviews have described ruthenium compounds as promising anticancer agents [11,12,13]. A variety of molecules containing Ru(II) or Ru(III) metal-centers have been synthesized which exhibit cytotoxicity against cancer cells similar to the commercial drug cisplatin [12], but in contrast to platinum-base drugs, these organoruthenium compounds are not sensitive to resistance mechanisms and may have fewer toxic side effects, since they are metabolized via similar pathways as iron (Fe^2+^) in the body [13]. In a typical ruthenium complex the metal-center is surrounded by aromatic, or bidentate chelating ligands to produce stable three-dimensional coordination geometries of different shapes, sizes, functional groups, and pharmacological potential. In this regard, organoruthenium complexes represent one of the most exciting platforms in modern chemotherapy. Ruthenium complexes are also known for their ability to selectively coordinate histidyl imidazole nitrogens in proteins and at the N7 site of purine nucleotides in DNA. Therefore, because of their specific-binding to these structural features, novel ruthenium compounds may potentially be designed with site-specific tissue distributions *in vivo* [14]. Ruthenium may be the ideal coordinating metal center for dipeptidic ligands such as L-carnosine. Because of its polydentate functionality and potent antioxidant qualities, carnosine-ruthenium (II) chelation may provide unique opportunities for rationale-based drug design.

In previous studies [15,16,17], the utility of heteronuclear nuclear magnetic resonance (NMR) techniques to gain structural information on metal-binding sites of carnosine and other histidine-containing peptides was demonstrated. Two-dimensional NMR spectra in these and other studies revealed that the terminal amine and the imidazole nitrogen of carnosine were effective σ-donating sites [17,18]. In the present work, novel ruthenium-carnosine complexes were synthesized in aqueous solution and studied via ^1^H-, ^13^C-, and ^15^N-NMR spectroscopy. Moreover, in an attempt to correlate our experimental findings with theoretical predictions, three possible ruthenium-carnosine complex structures have been proposed and compared at the DFT level. To the best of our knowledge, the interaction between carnosine and the transition metal ruthenium has not yet been described. Resulting effects of ruthenium coordination on carnosine electronic structural and their interactions in aqueous solutions are proposed and discussed.

## 2. Results and Discussion

### 2.1. Preparation of the Complexes

**Scheme 1:**
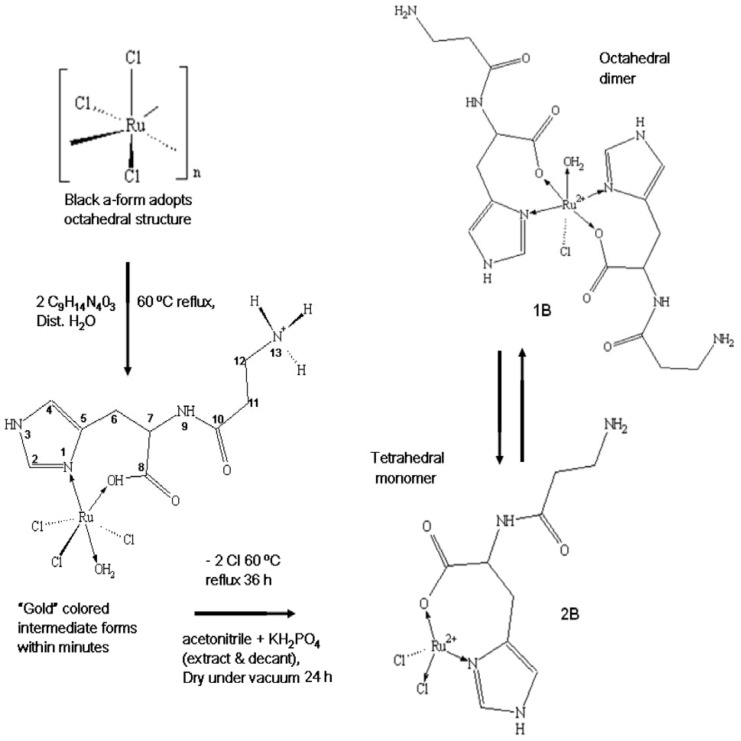
Preparation of Ru-Carnosine complexes.

Scheme 1 shows the stepwise preparation of complexes **1B** or **2B**. In the first stage equimolar amounts of L-carnosine and ruthenium chloride were washed with dichloromethane, then dried under vacuum for 24 h. The recrystallized starting materials were then refluxed at 60 °C in double-distilled water for 36 h. A black suspension resulted that was then vacuum-filtered to yield a reddish-brown solution containing the complexes. Aliquots of this solution were extracted with potassium hydrogen phosphate in acetonitrile to remove excess chlorides. The product forms a reddish-brown suspension which after decanting was allowed to settle; dried under vacuum; then re-dissolved in double-distilled water. The products formed sticky reddish-brown crystals with a melting temperature around 313 °C which are insoluble in organic solvents but soluble in distilled water. Sequential substitution of the chlorides from the metal precursor with carnosine may lead to formation of intermediate complexes but were not isolated in the current study.

Product samples were subjected to elemental analysis by energy-dispersive X-ray spectroscopy (EDX). The results of those experiments were converted to mean atomic ratios as listed in Table 1. The mean ratios divided by the number of each atom per ligand yields an approximate coordination number. The amount of free ligand in the product was estimated via excess nitrogen to give an approximate complexation efficiency of 41.7%.

**Table 1:** Physical and elemental analysis data summary.

Physical Characteristics						
Compound	Color		Yield		m.p.	Solubility
ligand	white				303–323 °C	Insoluble org., soluble aqueous
Complex 1B	reddish-brown		41.7%		313 °C	Insoluble org., soluble aqueous
Complex 2B	reddish-brown		41.7%		313 °C	Insoluble org., soluble aqueous
**Elemental Analysis** ****						
**Carbon (%)** ****		**Nitrogen (%)** ****		**Oxygen (%)** ****		**Ruthenium (%)** ****
47.78		24.77		21.22		0
27.08		14.03		12.02		25.32
35.62		18.46		18.45		16.65
**Mean Atomic Ratio (s.d.)** ****						
**Compound** ****		**Carbon:Ru** ****		**Oxygen:Ru** ****		**Nitrogen:Ru** ****
Ru-complex		18.5(3.8)		6.3(0.52)		19.1(1.1)
**Probable Coordination number [RuL_x_ClH_2_O] = Mean atomic ratio/atoms per ligand** ****						
Ru-complex		18.5(3.8)/9 = x = 2.1		6.3(0.52)/3 = x = 2.2		19.1(1.1)/4 = x = 4.8

### 2.2. Physical Characterization

#### 2.2.1. Differential Scanning Calorimetry/Thermogravimetric Analysis (DSC/TGA)

**Figure 1:**
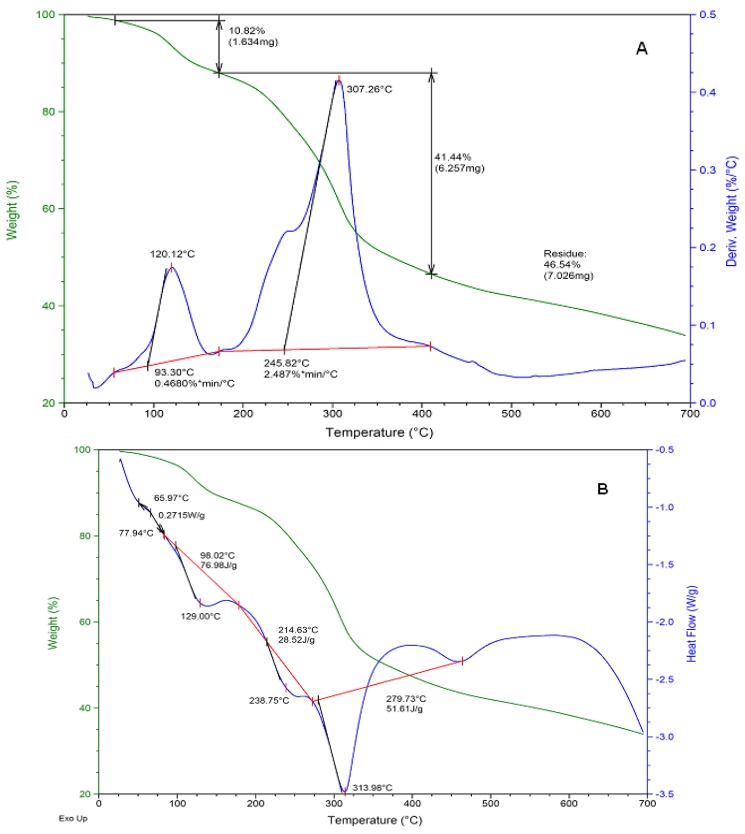
TGA (**A**) and DSC (**B**) of the Ru-carnosine complex.

Thermogravimetric and heat flow profiles of the ruthenium-carnosine complex were recorded in the temperature range 25–700 °C. Three major decomposition phases appear in Figure 1A, which begin with dehydration weight losses (~2%) occurring during the first stage. A second decomposition stage occurs from 93–120 °C in which loss of coordination water molecules (11%) takes place. This is followed by an apparent deligation process from 245–307 °C wherein the ligand (carnosine) is released from the metal center. This weight loss (41%) could be due to the decomposition of either dimeric or monomeric forms of the complex or the presence of both forms in the sample analyzed. A final decomposition stage occurs from 420–700 °C, after which a residual mass of approximately 47% remains.

The decomposition product consists predominately of metal oxides since ruthenium oxides form readily at these temperatures. The DSC thermogram (Figure 1B) illustrates several thermal transitions in the sample, with notable endotherms corresponding to dehydration (77.9 °C); release of coordination water (129.1 °C); and deligation of carnosine at (238.7 °C) are observed. The endotherm at 313.9 °C is in the range of the known fusion temperature (*i.e.*, *T_m_*) of the ligand. This peak appears broader than typical melting point transitions and is likely due to the presence of the ligand in different states of bonding or non-bonding. Beyond the melting point, thermal decomposition continues simultaneously with recrystallization showing exotherms about a glass transition (*i.e.*, *T_g_*) at 470 °C. Results show reasonable agreement with the formulae suggested from other physical property data in Table 2 and are indicative of structures proposed by DFT and spectroscopic methods.

**Table 2:** Thermal, electrochemical, and electronic spectroscopy data summary.

Thermal Analysis DSC/TGA for Ru-Complex							
Temp. Range (°C)	Weight loss (%)		Thermal event		Temp. Range (°C)		Thermal event
65–100	10.82		dehydration		50–80		dehydration
103–175	1.0		solvolysis		129		*Tg*
180–307	41.44		deligation		238		*Tg*
307–320	<1.0		decomposition		313		*Tm*
320–700	<1.0		decomposition		460		recrystallization
**Electrochemical Analysis for Carnosine and Ru-Complex** ****							
**Compound** ****	E_pa_ ****		E_pc_ ****		E_p1/2_ ****		ΔE_p_ ****
Carnosine	1.8		−0.6		0.6		2.4
Ru-Complex	0.94		−0.56		0.19		1.5
**UV-Visible Absorbance/Molar extinction coefficients** ****							
**Compound** ****		**Wavelength in nanometers (cm^−1^M^−1^)** ****					
L-carnosine		265 (1790 ^a^, 3886)		214 (7517)		209 (8350)	
Ru-complex		469 (726)		323 (1609)		215 (3573)	
Ru-complex ^b^		469 (726)		323 (1865)		222 (4677)	

^a^ See reference [36]; ^b^ Baseline corrected spectra.

#### 2.2.2. Cyclic Voltammetry

Electrochemical properties of the compounds were studied by cyclic voltammetry in phosphate buffer (pH 7.0) across a potential range of −2.0 to +2.0 Volts *versus* a SCE reference electrode using a scanning rate of 0.1 V/s. Both the ligand and complexes exhibited well-defined redox waves and it is assumed that all processes occurring at the electrodes were diffusion controlled. Carnosine had its characteristic voltammetric profile, giving a single anodic peak at +1.43 V which is indicative of reduction at one or both of its carbonyl groups. In contrast, the ruthenium complex voltammogram shows an irreversible oxidation peak at +0.94 V which may be assigned to the metal-center with its corresponding reduction potential at −0.54 V.

**Figure 2:**
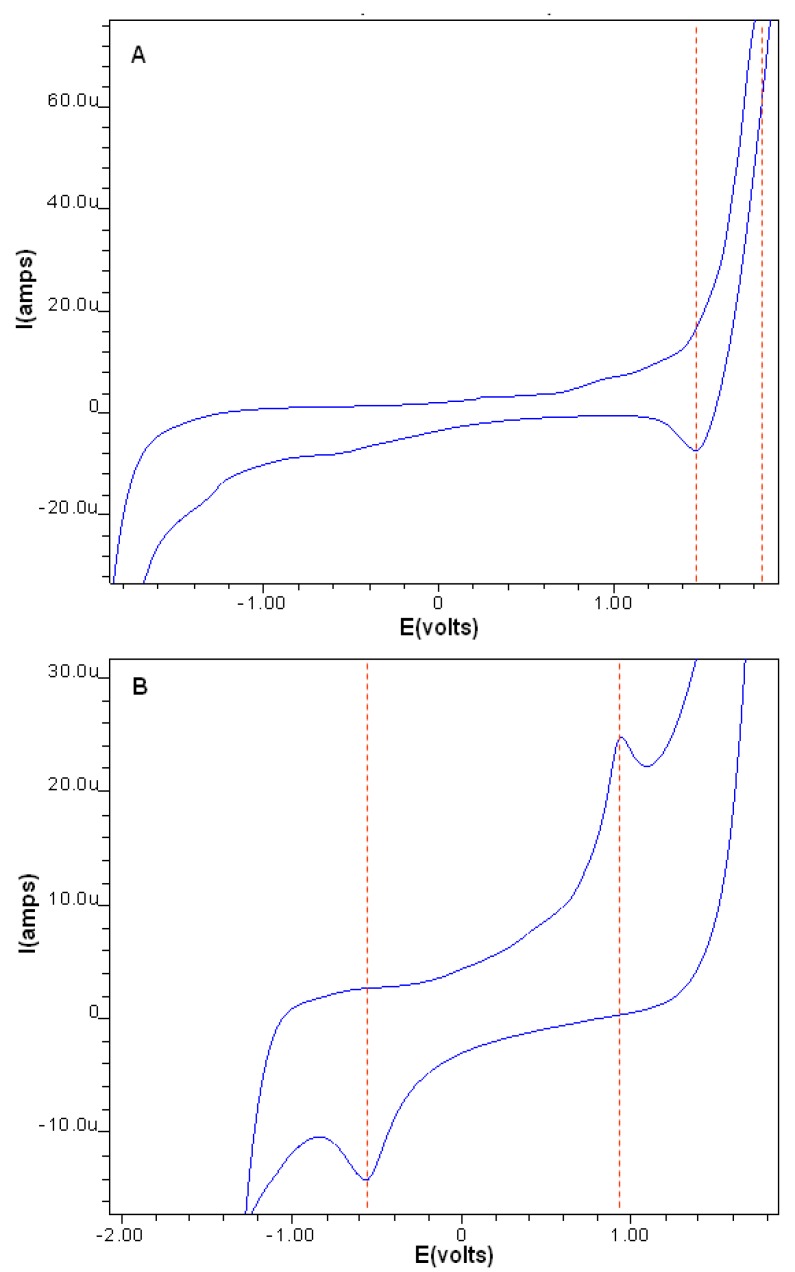
Cyclic voltammograms of carnosine (**A**) and the Ru-carnosine complex (**B**).

Reasonable agreement with previously published values for the reduction potentials of carnosine [19], as well as, the oxidation potentials of the ruthenium complex [20] have been cited. Peak separation potential ΔEp (1.5), however, is larger than typical Nernstian values for one-electron transfers, but this commonly observed in irreversible coordination compounds of this type [21].

Ruthenium oxidation in the complex may be facilitated by the σ-donating ability of the imidazole nitrogens which by stabilizing the Ru^+3^ oxidation state, support electron transfer. Opposing effects on the Ru^+2^/Ru^+3^ redox couple should also be mentioned since ligands containing carbonyl oxygen as donor atoms tend to stabilize the lower oxidation state; this is due to their π-acceptor ability which makes oxidation more difficult. We rationalize these observations by comparing our E_1/2_ values with other organoruthenium complexes which were found to be lower in magnitude [20,22,23].

### 2.3. Structure Elucidation

#### 2.3.1. Electronic Spectroscopy

**Figure 3:**
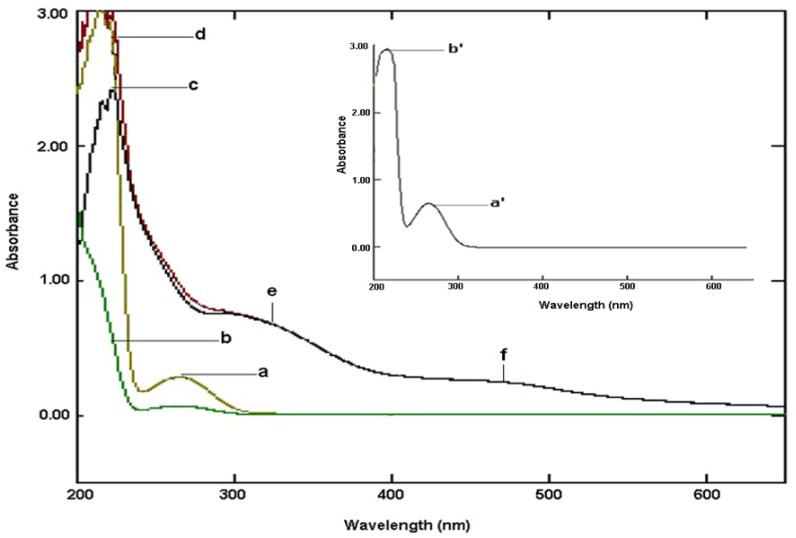
UV-Vis absorption spectrum of carnosine (0.88 mM **a’**, **b’**) and (0.42 mM **a**, **b**) and its ruthenium complex (**c**, **d**, **e** and **f**).

The UV-Vis absorbance spectra of ruthenium-carnosine complexes were obtained in distilled water (pH 6.9) at 25 °C. Figure 3 shows the electronic absorption spectrum (nm) of 0.88 mM L-carnosine (insert), with absorbance bands observed at 264.5 (**a’**), 214 (**b’**), and 209 (**b’**) which can be assigned to n-π*, π-π*, and π-π* respectively [24,25,36]. At low concentrations (0.07 mM) the peak at 265 nm of carnosine virtually disappears leaving only the broad band around 214 nm. In contrast to the dipeptide alone, the 0.42 mM product complexes were characterized by two shallow peaks at 323 nm (**e**) and 469 nm (**f**), representing ligand to metal charge transfer (LMCT), possibly promoted by dissociation of a proton from the imidazole ring nitrogen [26,27,32], and a high intensity band centered around 215 nm (**d**). However, when this spectrum was baseline corrected using equimolar L-carnosine as a blank, a peak at 222 nm (**c**) emerges in the spectrum while all other absorbances above 300 nm remained unchanged. This transition may again be associated with imidazole proton dissociation or changes in intraligand π-π* transitions after metal chelation. A summary of the electronic transitions of carnosine and its ruthenium complex is given in Table 2.

#### 2.3.2. Infrared Spectroscopy

**Figure 4:**
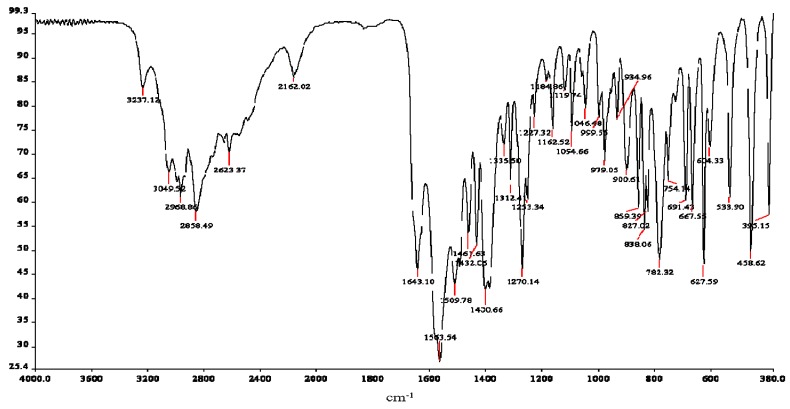
ATR-IR spectrum of L-carnosine.

**Figure 5:**
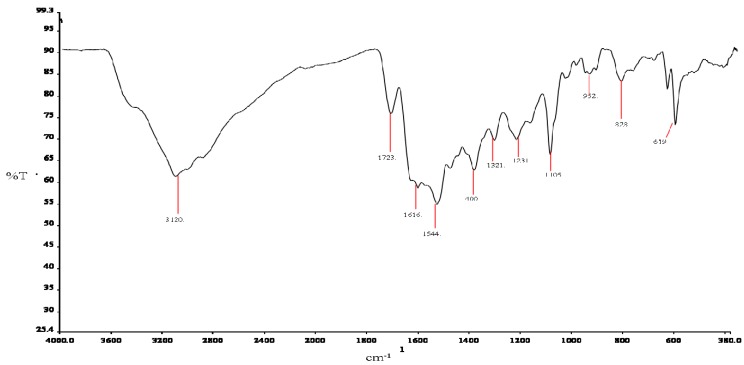
ATR-IR spectrum of Ru-carnosine Complex

The vibrational spectra provided an abundance of structural information regarding the chelation dynamics of carnosine (Figure 4) and its ruthenium complex (Figure 5). However, many of the absorbances for the complex or the free ligand were difficult to assign because of intramolecular proton displacements and conformational changes after binding of ruthenium, even at large distances from the metal center. The availability of heteronuclear NMR data and published vibrational assignments has made it possible, in most cases, to propose assignments for the groups directly involved in coordination. A summary of the infrared vibrational assignments is given in Table 3.

**Table 3:** Infrared vibration assignments for L-carnosine and Ru-carnosine complex.

Atom#	Carnosine *V*(cm^−^^1^)	Assignments	Atom#	Ru-complex *V*(cm^−^^1^)	Assignments
N13	3237	ν_a_(NH_3_)^+^	-----	-----	-----
N13	3049	ν_a_(NH_3_)^+^	-----	-----	-----
-----	-----	-----	N3	3120	ν_a_ (N-H) *
-----	-----	-----	C1, N3	1723	ν(N_1_=C_2_) *
-----	-----	-----	-----	-----	-----
C10	1643	Amide l	C8	1616	Amide l
C4, 5	1563	ν(C_4_=C_5_) *	-----	-----	-----
-----	-----	-----	C8	1544	v_a_(COO)^−^
imidazole	1461	stretching *	-----	-----	-----
N3, C11,12	1432	δ(N-H) *, δ(CH_2_)	-----	-----	-----
C8	1400	ν_s_(COO)^−^	C8	1400	ν_s_(COO)^−^
C2, 4, 7	1335	ν(C-N) + breathing *	-----	1321	ν(C-N) + breathing *
C2, 4	1312	ν(C-N) + breathing *	-----	-----	-----
imidazole	1270	Breathing *	-----	-----	-----
N1, C2, N3	1227	ν(NCN) * + δ(N-H) *	N1, C2, N3	1231	ν(NCN) * + δ(N-H) *
N1, C2, N3	1162	ν(NCN) * + δ(N-H) *	-----	-----	-----
C7	1095	δ(C-H) *	C7	1105	δ(C-H) *
C2, 4	979	δ(C-H) *	C2, 4	952	δ(C-H) *
imidazole	859	deformation *	-----	-----	-----
N13	838	NH_3_^+^ deformation	N13	828	NH_3_^+^ deformation
C8	625	δ(COO)^−^	C8	619	δ(COO)^−^

ν = stretching; ν_a_ = asymmetric stretching; ν_s_ = symmetric stretching; δ = in-plane bending; * = imidazole ring.

The most important vibrations in the high-frequency region included stretching modes (3,237 cm^−1^ and 3,049 cm^−1^) for the protonated terminal amine ν_a_(NH_3_)^+^ of the free ligand. While these bands were distinctly represented in carnosine, their absorbance in the complex appears subdued by hydration peaks and hydrogen-bonding. A N-H stretching mode vibration at 3,120 cm^−1^ dominates the region after coordination, probably due the “pyrrole-like” or “tau” nitrogen of the imidazole ring. The intensity of neither of the bands in the region changed significantly; although some of the bands were affected by complexation with the metal, none of these groups participated directly as donor atoms.

The carboxylate stretching modes in the complex and free ligand are unusual, but comparable with observations reported in previous studies [28,29]. Asymmetric stretching modes did not appear in the spectrum of carnosine, but do so after coordination. This may be caused in part by overlapping ring vibrations at 1,563 cm^−1^ (ν(C_4_=C_5_) * and ν(C=N) *), as well as enhanced asymmetry of the carboxylate via its interaction with ruthenium [9,30]. Another unusual feature of carnosine-transition metal interactions involves the displacement of amide I band vibrations without direct interaction with the metal center. It is possible that this displacement is a consequence of deprotonation of the proton from the amide nitrogen, causing a lowered carbonyl frequency (1,643 to 1,616 cm^−1^) and appearing as a weak shoulder. Thus, although not directly involved in the coordination, the amide l band is nevertheless affected by the interaction with ruthenium.

The correlations between imidazole vibration bands and this group’s involvement in complexation are well established. Ring vibration modes at 979 cm^−1 ^decreased in frequency and intensity to 952 cm^−1^ due to metal chelation. Coordination furthermore has an effect on the tautomeric equilibrium of the ring; *i.e.*, binding of ruthenium by one of the two ring nitrogen atoms, limits the site of protonation to the other. Therefore, notable changes in the spectrum of the complex in stretching mode vibrations of the imidazole nitrogens (ν(NCN) * and δ(N-H) *) were observed at 3,120, 1,432, and 1,227 cm^−1^; with weakening or disappearance of vibrations at 1,461, 1,432, 1,312, and 1,270 cm^−1^. A possible consequence of changes in ring tautomeric equilibrium [28,37] after complexation is the emergence of a stretching vibration ν(N_1_ = C_2_) * at 1,723 cm^−1^. This peak occurs after coordination and is likely associated with the “imine-like” or (pi) nitrogen of imidazole. Participation of nitrogen as a donor atom in the coordination system weakens the imine-linkage so that its frequency is substantially lowered.

In the 1,400–620 cm^−1^ frequency region imidazole ring breathing and stretching vibrations are clearly shifted to higher wave number medium intensity bands, in agreement with participation of this moiety in complexation. The behavior of both the carboxylate stretching modes is also interesting. Symmetric stretching modes ν_s_(COO^−^) were completely unchanged, but participation in coordination is confirmed by bending mode vibrations δ(COO^−^) which were lower in frequency (628 to 619 cm^−1^) and intensity in the complex. While the asymmetric and symmetric vibration bands were practically unchanged the involvement of the carboxylate group in coordination is indicated from displacement of the δ(COO^−^) mode to lower wave number values after coordination. These observations may suggest some maintenance of ionic character during the metal-O interaction and that deprotonation of the acid may be stabilized via hydrogen-bonding [28,29,30]. Analysis of the lower-frequency region did not identify any vibrations likely to be association with ruthenium-ligand.

#### 2.3.3. ^1^H-, ^13^C- and ^15^N-NMR Studies

**Figure 6:**
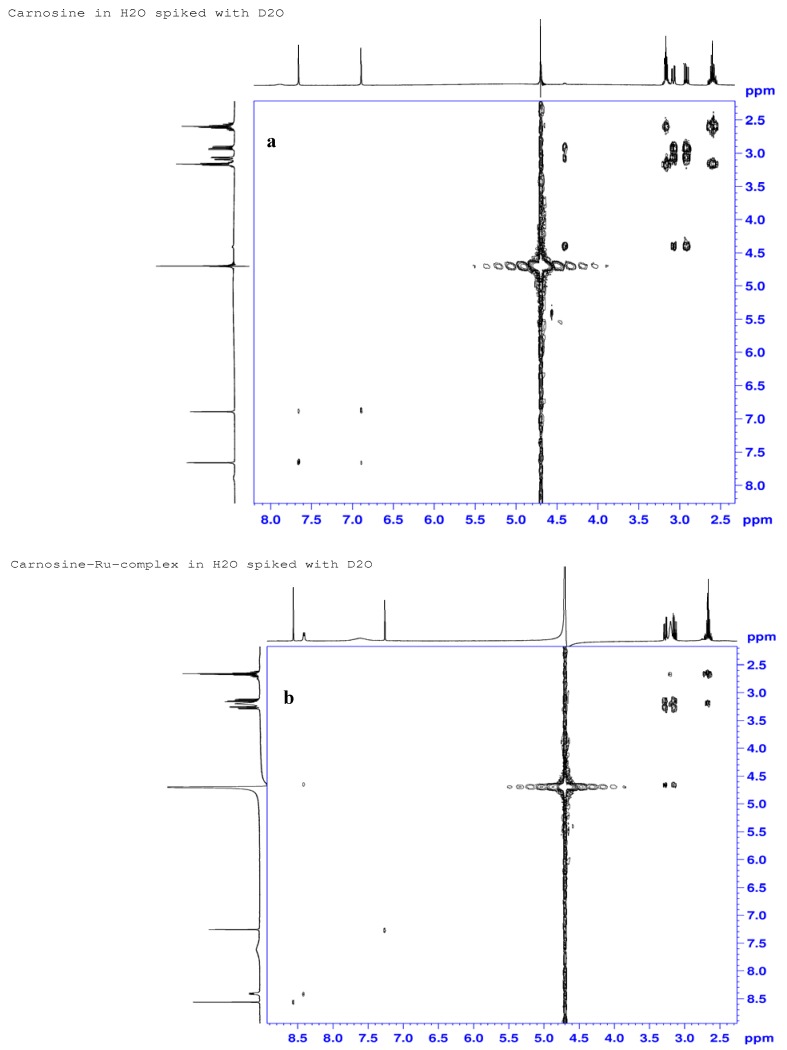
^1^H-NMR COSY Spectra for L-carnosine (**a**) and Ru-carnosine complex (**b**).

Proton NMR COSY spectra of carnosine and its ruthenium complex are shown in Figure 6a and 6b. Observed peaks for carnosine occurred at δ 2.60, 2.92, 3.08, 3.16, 4.40, 6.89, 7.66 and 7.89 ppm and are representative of the following protons: β’-ala-methylene (H12), β_S_-his-methylene (H6), β_R_-his-methylene (H6), α’-ala-methylene (H11), α-his-methine (H7), imidazole ring methine (H4), and imine hydrogen (H2), and amide N-H (H9), respectively. Notable changes in the chemical shifts for the H7 (δ 4.4 to 4.65 ppm), H2 (δ 7.66 to 8.56 ppm) and H4 (6.89 to 7.26) protons indicate significant deshielding of the carboxylate (COO^−^) and imidazole groups after interaction with the metal. Proton signals at positions H11 and H12 did not show any appreciable changes, suggesting non-participation of the carnosine N-terminus (β-alanine) in the complexation. Similarly, the characteristic doublet (*J* = 7.8 Hz) of H9 at δ 8.41 ppm in the proton spectrum of the complex excludes involvement of the amide at N9. The signal corresponding to H7 was not visible in the spectrum of the complex due to overlap with a signal from solvent water; however, a small cross-peak in the COSY spectrum (Figure 6b) at 4.65 ppm × 8.41 ppm confirmed its presence in the complex. Negative RCIS values observed for ring protons after complexation due to ring-current anisotropy effects are also present since coordination with the metal places these hydrogens over the shielding plane of the aromatic ring. The proton at position 2 exhibited the greatest downfield shift (11.7%) because of ligand–to-metal σ-donation by the adjacent nitrogen. Both H6 (−7.5 and −6.5%) and H7 (−5.7%) protons were each affected by complexation, although these effects probably originate from conformational changes and their proximity to the ring.

**Figure 7:**
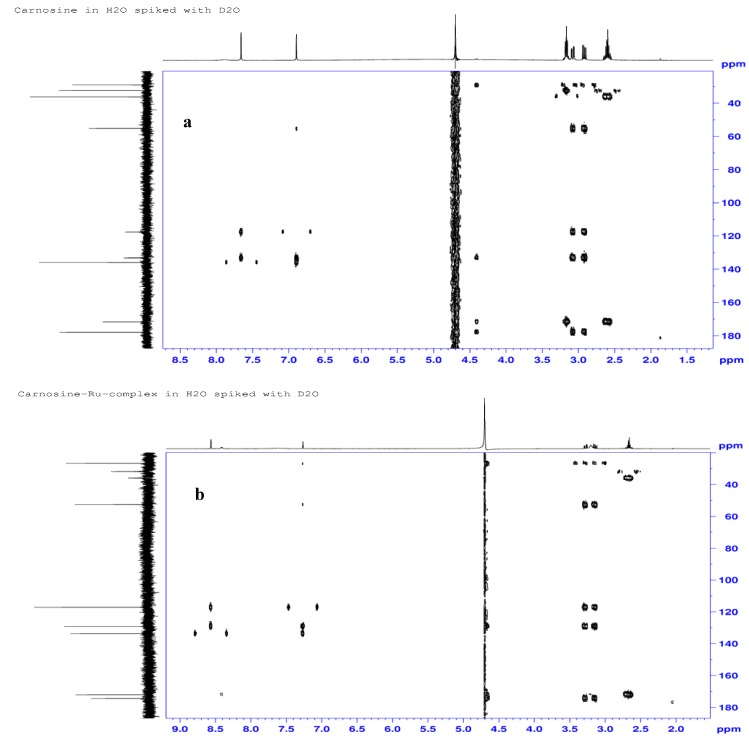
^1^H-^13^C-NMR correlation (gHMBC) spectra for L-carnosine (**a**) and Ru-carnosine complex (**b**).

The ^13^C-NMR spectra of L-carnosine (Figure 7a) and the prepared complex (Figure 7b) revealed that five of the nine resonances in the complex are shifted upfield to different extents, in the order C4 (4.1 ppm) > C8 (3.4 ppm) > C7 (2.6 ppm, 2.3 ppm) > C6 (2.5 ppm, 2.4 ppm) > C2 (2.3 ppm). The observed chemical shift changes not only suggest involvement of the carboxylate and the imidazole groups, but also illustrate the effects of ruthenium coordination on distant atoms due to its ability to induce conformational changes in the ligand. Small changes observed in chemical shifts for C11 (0.4 ppm) and C12 (0.6 ppm) were in good agreement with the corresponding small proton shifts (H11, H12). The presence of double carbon signals for C6, C7 and C10 in the carbon spectra also suggest ligation by two carnosine molecules in the formed complexes.

Most of the carbon RCIS values correlated well with proton shift changes in magnitude, but not in sign. The largest RCIS for carbon also occurred at C6 (8.6 and 8.3%) and C7 (4.9; 4.7%) and are likewise due to some degree of steric compression by the metal center. Upfield shifts in these aliphatic carbons further suggest that they undergo some energy-minimizing conformational changes upon complexation that the carbon atom C5 cannot. Aliphatic carbons C11 and C12 were only mildly effected, which may be caused by their proximity to a terminal ammonium ion alone.

**Figure 8:**
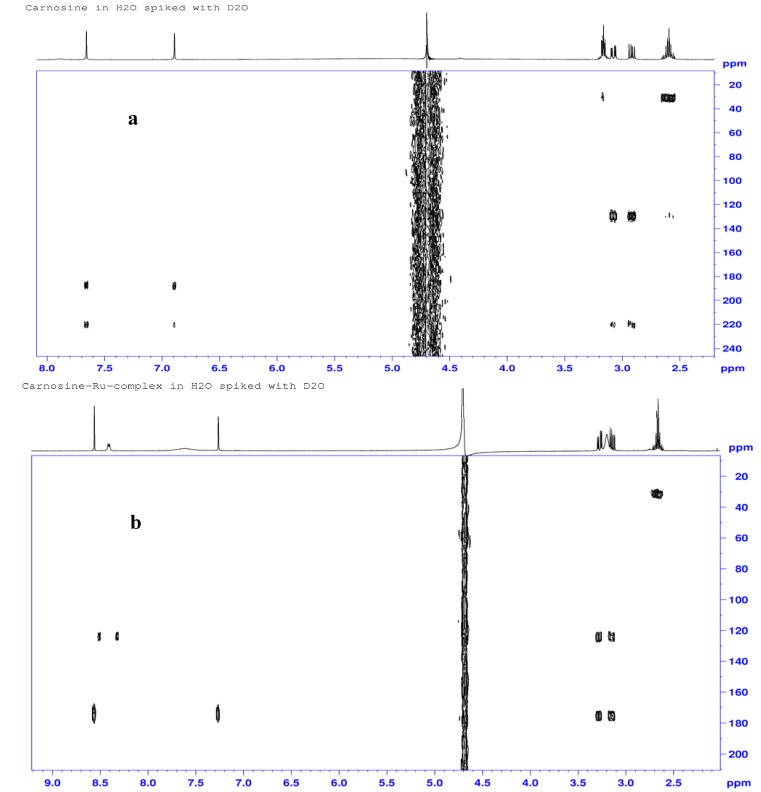
^1^H-^15^N NMR correlation (gHMBC) spectra for L-carnosine (**a**) and Ru-carnosine complex (**b**).

Imidazole carbons C2 (1.7%), C4 (3.1%), and C5 (0.4%) were not as sensitive to metal-interactions as the hydrogen or nitrogen atoms of the ring. But according to literature reports [31,32,33], the relative contribution of ring-current anisotropy is less responsive in ^13^C-NMR than in ^1^H-NMR chemical shifts. Ring carbon C4, however, was affected more than C2, in agreement that the notion that the position 4 methine would be the most sensitive carbon to the σ–donation effects of the metal. The large change at proton H2 was regarded as a ring-current distortion anomaly and not a coordination effect. A main contributor to the ^13^C-NMR RCIS values appears to be ligand-to-metal σ–donation, which generally leads to positive RCIS values for the ring carbons. The modest RCIS for carboxylate (1.9%) is somewhat smaller than would be expected after coordination, but the presence of other H-bonding oxygens may have subdued any metal-induced shift so that only a small difference was observed.

No significant change in chemical shift of the amide-carbonyl spectra is reported and the RCIS value here was essentially zero. Table 4 lists the NMR chemical shift assignments and RCIS values for each of the nuclei. Three of the ^13^N resonances undergo upfield shifts, *i.e.*, N9 (δ 129.5 to 124.1 ppm), N1 (δ 220.7 to 175.3) and N3 (δ 188.5 to 175.2 ppm). Distinct upfield shifts consistent with shielding of both ring nitrogens N1 (45.4 ppm) and N3 (13.3 ppm) are observed as shown in the ^1^H-^15^N gHMBC spectra (Figure 8a and b).

**Table 4:** NMR chemical shift assignments.

Atom#	Carnosine (ppm)	Ru-complex (ppm)	%Delta δ (ppm)	Assignments
**H7**	4.40	4.65	−5.7	α-His-methine
**H6**	2.92; 3.08	3.14; 3.27	−7.5; −6.5	β_S_; β_R_ -His-methylene
**H9**	7.89	8.41	−6.6	Amide N-H, H-bonding
**H2**	7.66	8.56	−11.7	Imine hydrogen
**H4**	6.89	7.26	−5.37	Ring methine
**H11**	3.16	3.2	−1.9	β-Ala-methylene
**H12**	2.60	2.66	−2.3	α-Ala-methylene
**C8**	177.9	174.5	1.9	Carboxylate
**C7**	55.3	52.6; 52.7	4.9; 4.7	α-His-methine
**C6**	29.0	26.5; 26.6	8.6; 8.3	β-His-methylene
**C2**	135.9	133.6	1.7	Imine carbon
**C4**	133.2	129.1	3.1	Ring methine carbon
**C5**	117.6	117.1	0.4	Ring carbon atom
**C10**	171.7	172.0; 172.0	−0.1	Amide carbonyl
**C11**	36.1	35.7	1.1	β-Ala-methylene
**C12**	32.5	31.9	1.8	α-Ala-methylene
**N9**	129.5	124.1	4.2	Amide nitrogen
**N3**	188.5	174.0	7.7	Pyrrole nitrogen
**N1**	220.7	175.3	11.5	Imine nitrogen
**N13**	30.9	31.4	−1.6	NH_3_

The large change of the (N1) nitrogen signal is consistent with the shielding effects of coordination with a heavy metal. This “imine-like” or (pi) nitrogen of the ligand also presented the largest RCIS at 11.5%. The coordinated nitrogens are involved to some degree in π-backdonation, as reported by others [33,34,35] which is quite possible in ruthenium coordination. The asymmetric nature of the relative shift changes at N3 (7.7%) and N1 (11.5%) support this and furthermore indicate participation of the “imine-like” nitrogen rather than the “pyrrole-like” nitrogen during metal-coordination. The amide nitrogen N9 undergoes a modest RCIS (4.2%), but this was accompanied by a larger H9 value (−6.6%). This shielding/deshielding response of different nuclei at the same position is thought to be due to conformational effects of H-bonding or interaction with OH-radicals rather than participation at the metal center [36]. Larger conformational changes in the histidine segment than in the central (amide region) of the carnosine molecule are implied during complex formation. The small downfield shift from δ 30.9 to 31.4 ppm for (N13) effectively ruled out its participation in metal coordination, and consistent with results obtained from the ^1^H and ^13^C spectra.

### 2.4. Computational Methods

**Figure 9:**
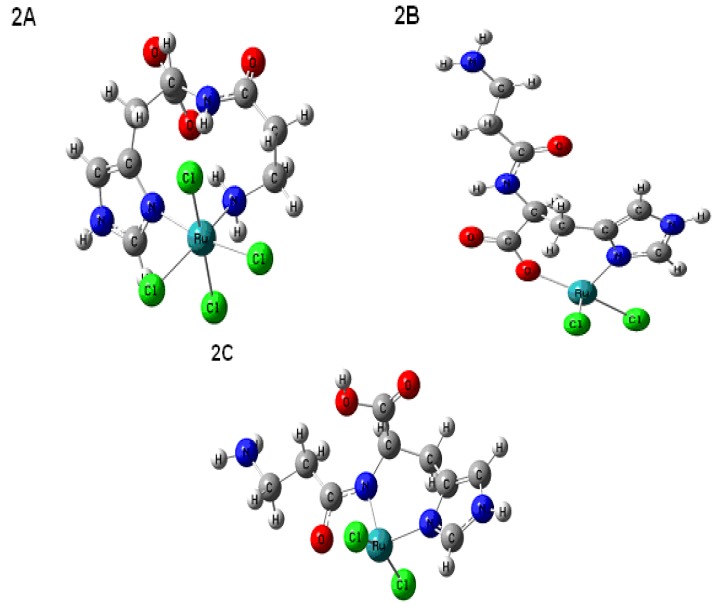
Low energy conformations of different proposed structures for Ru-carnosine complex.

Based on experimental results, three possible monomeric geometries **2A**, **2B**, **2C** for the complexes were investigated at (DFT) B3LYP level with 6-31G*/lanl2dz mixed basis set. Optimized geometries of each proposed structure are diagrammatically represented in Figure 9, and the computed results for NMR (^1^H and ^13^C) and IR vibrational frequencies are summarized in Table 5.

All optimized structures (**2A**–**2**C) are true ground state minima, verified by second derivative frequency calculations. A closer inspection of the results of Table 5 reveals that the calculated proton chemical shifts for structures **2B** and **2C** are in good agreement with the experimental measurements. Complex **2A**, on the other hand, exhibits an appreciable departure from the experimental assignments, especially for C4 (30 Å) and C5 (10 Å) atoms. The results are clearer from the proton shifts of complex **2B**, which satisfactorily reproduced the experimental proton chemical shifts better than **2C**. Structure **2A** presents a larger disagreement with experimental measurements particularly for H4 (15.4) and H9 amide (16.3) protons. Each proton was considered non-equivalent in the Gaussian calculations irrespective of its molecular environment which is why no any splitting pattern is obtained in the computed proton spectrum of the complexes. Furthermore, the computed harmonic vibrational frequencies (in cm^−1^) for complex **2B** showed good agreement with the experimental values. The characteristic symmetric stretching mode of carboxylate (COO^−^) functionality at 1,408 cm^−1^ and amide carbonyl (C=O) at 1,638 cm^−1^, clearly supported the structure **2B** over other possible geometries [37]. Finally, the measured interatomic distances between the donor atoms of carnosine and ruthenium in complex 2B (Ru-Cl = 2.33, Ru-Cl = 2.38, Ru-N-imida = 2.01, Ru-O = 1.93) were also found to be comparatively shorter than those present in the complexes 2A (Ru-N-imida = 2.21; Ru-N-amine = 2.1; Ru-Cl = 2.41; Ru-Cl = 2.43; Ru-Cl = 2.42; Ru-Cl = 2.36 or 2C (Ru-N-imida = 2.03; Ru-N-amide = 1.94; Ru-Cl = 2.40; Ru-Cl = 2.41). These results also suggest that tetrahedral complex **2B** including its carboxylate and imidazole moieties is more compact and more stable than complexes **2A** or **2C**.

**Table 5:** Experimental and DFT predicted NMR and IR spectral data.

Experimental δ_C_	Predicted δ_C_ 2A	Predicted δ_C_ 2B	Predicted δ_C_ 2C
133.57	164.7	131.6	134.1
129.06	140	130.5	132.2
117.1	110.8	115.2	110.6
26.57	29.3	25.1	23.4
52.7, 52.6	54.2	55.1	55
174.5	180.1	176	184.5
172.0, 171.96	177.6	172.7	175
35.7	41	36.3	32.1
31.88	29.4	33.2	30.4
**Experimental δ_H_** ****	**Predicted δ_H_ 2A** ****	**Predicted δ_H_ 2B** ****	**Predicted δ_H_ 2C** ****
8.6(s)	25.8	8.8	6.3
3.2–3.3(m)	4.5	3.33	3.7
3.1–3.3(m)	4.22–4.42	3.25–3.32	3.1–3.6
7.4	8.8	6.6	5.6
7.6	16.3	7.9	--
2.6–3.4(m)	1.1–5.9 (CH2)	1.2–2.5 (CH2)	1.3–2.4(CH2)
8.4(s)	9.5	7.3	6.85
	6.4		5.8
**Experimental cm** **^−1^** ****	**Predicted cm** **^−1^ 2A** ****	**Predicted cm** **^−1^ 2B** ****	**Predicted cm** **^−1^ 2C** ****
1723(C=N)			
-----	1692	1647 (acid C=O)	1704
1647	1671	1638	1506
1544			
1400	-------	1408	-----
1321	1331	1322	1351
1237	1193	1228	----
1105	1121	1114	1243

## 3. Experimental

### 3.1. Materials

L-Carnosine (2-[(3-aminopropanoyl)amino]-3-(1H-imidazol-5-yl)propanoic acid) (226.2 g/mol, T_m_ > 253 °C, 99% pure) and ruthenium chloride (octahedral, black-α form, 207.43 g/mol, T_d_ > 500 °C, 99% purity) were purchased from Sigma-Aldrich (Gauteng, South africa) and used as supplied. Purified water used to prepare the complexes was produced in-house with a Milli-Q water purification system (Millipore Corp., Johannesburg, South Africa). All other chemicals used in the preparation of the complexes were reagent grade. Freeze-dried ruthenium complexes were analyzed for atomic distribution by energy-dispersion x-ray spectroscopy (EDX), using JEOL-JSM 7500F (JEOL LTD., Herts, England) operating at an accelerating voltage of 1 kV.

### 3.2. Synthesis of the Complexes

#### 3.2.1. Characterization of L-Carnosine (2-[(3-Aminopropanoyl)amino]-3-(1*H*-imidazol-5-yl)propanoic Acid)

White crystalline solid; insoluble in organic solvents, soluble in distilled water; melting point = 253 °C; elemental analysis: calculated C(47.78%) H(6.24%) N(24.77%) O(21.22%); Molecular Formula: C9H14N4O3; Formula Weight: 226.2324; Percentage yield: ND; UV-Vis (nm, dist.H_2_O): 265, 214, 209; IR (cm^−1^, solid): 3237, 3049, 2969, 2858, 2623, 2162, 1643, 1561, 1461, 1431, 1409, 1400, 1312, 1270, 1253, 1227, 1184, 1162, 1120, 1054, 1046, 999, 979, 935, 900, 859, 838, 827, 782, 754, 691, 667, 627, 601, 533, 459, 395; ^1^H-NMR (ppm; H_2_O): δ 2.60 (m, 2 Hs, H12); 2.92 (dd, *J* = 8.7, *J* = 15.1, 1 Hs, H6); 3.08 (dd, *J* = 4.7, *J* = 15.1, 1 Hs, H6); 3.16 (m, 2 Hs, H11); 4.40 (dd, *J* = 4.4, *J* = 8.2, 1 Hs, H7); 6.89 (s, 1 Hs, H4); 7.66 (d, J = 0.9, 1 Hs, H2); 7.89 (small broad singlet, H-bonding). ^13^C-NMR (ppm; H_2_O + D_2_O): δ 29.0 (t, 1 Cs, C6); 32.5 (t, 1 Cs, C12); 36.1 (t, 1 Cs, C11); 55.3 (d, 1 Cs, C7), 117.6 (d, 1 Cs, C5); 133.2 (s, 1 Cs, C4); 135.9 (d, 1 Cs, NCHNH); 171.7 (s, 1 Cs, C10); 177.9 (s, 1 Cs, C8). ^15^N-NMR (ppm; H_2_O + D_2_O): δ 30.9 (N13); 129.5 (N9), 188.5 (N3); 220.7 (N1).

#### 3.2.2. Synthesis of the Complexes

Ruthenium carnosine coordination complex was obtained by reacting equimolar amounts of ruthenium chloride (3.3 mmol, 0.68 g) and L-carnosine (2.84 mmol, 0.64 g) at 60 °C in double distilled H_2_O (90 mL) for 36 h. The product mixture was then extracted (v/v 10:1) with KH_2_PO_4_ (50 g) in acetonitrile (250 mL) to remove excess free chloride. In this mixture the [**1B** and **2B**] complex appears as a reddish-brown suspension which after filtration was allowed to settle for 24 h. The product complexes were then isolated from acetonitrile by decanting/evaporation. As some of the complexation product was precipitated onto the solid-phase phosphate salt the process only yields approximately 41.7% after evaporation of the organic solvent. The reddish-brown product formed sticky crystals with a melting temperature around 313 °C. Colour: reddish-brown amorphous solid; Solubility: insoluble in organic solvents, soluble in distilled water; Melting point = 313 °C; Elemental Analysis (**1B**): Calculated C(35.62%) H(4.98%) Cl(5.84%) N(18.46%) O(18.45%) Ru(16.65%); Molecular Formula: C18H30ClN8O7Ru; Formula Weigh%: 607.00; Percentage yield: 41.7%; Elemental Analysis (**2B**): Calculated C(27.08%) H(3.79%) Cl(17.76%) N(14.03%) O(12.02%) Ru(25.32%); Molecular Formula: C9H15Cl2N4O3Ru; Formula Weight: 399.21; Percentage yield: 41.7%; UV-Vis (nm, dist.H_2_O): 469, 323, 222; IR (cm^−1^, solid): 3237, 3120, 1724, 1616, 1544, 1400, 1322, 1237, 1106, 619; ^1^H-NMR (ppm; H_2_O): δ 2.66 (m, 2 Hs, H12); 3.14 (dd, *J* = 15.5, *J* = 8.2, 1 Hs, H6); 3.20 (br s, 2 Hs, H11); 3.27 (dd, *J* = 15.5, *J* = 5.5, 1 Hs, CH2-CqN); 4.65 (not visible in proton spectrum, visible in COSY spectrum, 1 Hs, H7); 7.26 (s, 1 H, H4); 7.61 (br s, 2 Hs, H-bonding); 8.41 (d, *J* = 7.8, H9); 8.56 (d, *J* = 1.2, H2). ^13^C-NMR (ppm; H_2_O + D_2_O): δ 26.5 (t, 1 Cs, C6); 26.6 (t, 1 Cs, C6); 31.9 (t, 1 Cs, C12); 35.7 (t, 1 Cs, C11); 52.6 (d, 1 Cs, C7); 52.7 (d, 1 Cs, C7); 117.1 (d, 1 Cs, C5); 129.1 (s, 1 Cs, C4); 133.6 (d, 1 Cs, C2); 171.96 (s, 1 Cs, C10); 172.04 (s, 1 Cs, C10); 174.5 (s, 1 Cs, C8). ^15^N-NMR (ppm; H_2_O): δ 31.4 (N13); 124.1 (N9), 174.0 (N3); 175.3 (N1).

### 3.3. Physical Measurements

#### 3.3.1. Differential Scanning Calorimetry/Thermogravimetric Analysis (DSC/TGA)

DSC/TGA thermograms of milled and un-milled drug samples were obtained using a TA Instruments SDT-Q600 thermal analyzer (AMS Laboratory Technologies Ltd., Cape Town, South Africa) equipped with a computerized thermal data analyzer. Drug samples were weighed (5–10 mg) in flat-bottomed aluminum pans and heated from 25 to 700 °C at 5 °C/min under a nitrogen purge gas flowing at a rate of 5 mL/min.

#### 3.3.2. Cyclic Voltammetry

All electroanalytical measurements were performed with a 797 VA Computrace (Metrohm, Herisau, Switzerland). A three electrode system of 3 mm diameter rotating glassy carbon electrode (GCE), reference electrode made of Ag/AgCl (saturated AgCl, 3 M KCl), and the platinum wire auxiliary electrode was used. A pH meter, Crison Micro pH 2000, from Crison Instruments (Gauteng, South Africa) was used to adjust the pH of the buffer solution. All working solutions and the buffer were prepared with deionized water from an Aqua Max^TM^ Basic 360 Series water purification system from Trilab (Durban, South Africa). Software provided with the equipment enabled automatic peak evaluation (peak potential). Sodium dihydrogen phosphate dihydrate (NaH_2_PO_4_·2H_2_O) were obtained from Capital Lab Supplies (Durban, South Africa). Nitrogen of 99.9% purity was obtained from AFROX (Durban, SA). Alumina powder ≤3 µm was supplied by Metrohm (Durban, South Africa). The Glassy Carbon Electrode (GCE) surface is easily contaminated with the product of the electrode redox processes, hence it was renewed by polishing for 10 s with alumina paste (mixture of alumina and water) on a polishing cloth followed by rinsing with high-purity water and drying with nitrogen. Electrochemical cleaning was also performed by scanning 10 cycles in the potential range between −2.0 and 2.0 V in the presence of a supporting electrolyte. Voltammetric responses were recorded in 10 mL phosphate buffer pH 7.4. Potential was scanned at 0.1 V/s from −200 to 200 mV *vs.* SCE, after holding the electrochemical system for deposition at the initial potential for 120 s and purging for 300 s. The GCE was always thoroughly rinsed with distilled as described above after every single experiment.

#### 3.3.3. UV-Vis Spectroscopy

A Shimadzu 1800 UV/Vis spectrophotometer equipped with a 1 cm path-length cell was used to qualitative monitoring of complex formation. Spectra were recorded in the range of 200–800 nm for carnosine (neat) and Ru-complex by dissolving approximately 30 mg in 100 mL distilled water.

#### 3.3.4. Infrared Spectroscopy

FTIR spectra of the milled compounds were recorded on an Alpha Platinum ATR-IR spectrophotometer (Bruker, Bryanston, South Africa). The instrument was configured with a Pike ATR sample cell including a diamond crystal with a scanning depth up to 2 micrometers. Sample powders were applied to the surface of the crystal then locked in place with a “clutch-type” lever before measuring transmittance. Each of the spectra was collected in the range 4,000–400 cm^−1^ at 2 cm^−1^ resolution.

#### 3.3.5. Nuclear Magnetic Resonance Spectroscopy

^1^H-, ^13^C- and ^15^N-NMR spectra of carnosine and it’s ruthenium complex in solution were obtained using a Bruker Avance-III 500 (11.7 T) instrument operating at 500 MHz (1H), 125 MHz (^13^C), and 50 MHz (^15^N) using either a 5 mm BBO-Z probe or a 5 mm TBI-Z probe. Samples of carnosine and the ruthenium-carnosine complex dissolved in H_2_O were spiked with D_2_O as a field-frequency lock (Aldrich, 99.9% atom D) at ~10% by volume (~50 mL). Proton spectra were recorded using both WATERGATE and PRESAT pulse programs. Proton and carbon chemical shifts are reported in ppm (δ) downfield from tetramethylsilane (TMS) at δ = 0 ppm using the H_2_O/HDO signal (δ = 4.72 ppm) for proton shifts and using the lock frequency of the spectrometer for ^13^C shifts as secondary references. Coupling constants are reported in Hertz (Hz). Nitrogen chemical shifts were obtained from the projections of standard g-HMBC experiments and are reported in ppm (δ) downfield from a liquid ammonia at δ = 0 ppm using the lock frequency of the spectrometer as a secondary reference. Chemical shifts of the free and complexed ligand, along with their relative coordination-induced shifts (RCIS = [δ_ligand_ − δ_complex_] / δ_ligand_) are listed in Table 4.

### 3.4. Computational Methods

The starting structures of all complexes all monomeric (A-C) carnosine-ruthenium complexes were energetically minimized using the Forcite module of Materials Studio (MS). 19 Subsequently density functional theory (DFT) calculations at the B3LYP level were performed on the minimized complexes using lanl2dz effective core potential basis set for ruthenium and 6-31G* for the remaining atoms. All calculations were performed using the Gaussian 03 computer program [38]. Twenty equilibrium geometries, harmonic vibrational frequencies and nuclear magnetic resonance (NMR) chemical shifts were computed for each complex and compared with the experimental results.

## 4. Conclusions

The results of each of the spectrochemical experiments indicates stable bidentate complexes formed using two donor atoms of the ligand, namely, the carboxylate oxygen and imine-like nitrogen (N1) of the imidazole ring. On the basis of spectroscopic and computational evidence it is believed that in the prepared complexes, the tautomeric form present involves protonation of the “pyrrole-like” N(3) nitrogen. Electrochemical results confirm the +2 oxidation state for the metal-center and the thermogravimetric measurements on the deligation and residual masses of the complex are supportive of both dimeric and monomeric coordination geometries. Based on experimental results, three possible monomeric geometries (A-C) of carnosine-ruthenium complex were investigated at (DFT) B3LYP level with 6-31G*/lanl2dz mixed basis set. The characteristic symmetric stretching mode of the carboxylate (COO^−1^) functionality at 1408 cm^−1^ and the amide carbonyl (C=O) at 1,638 cm^−1^, clearly supported the structure 2B confusing with 2B-use consistent nomenclature over other possible geometries. The measured interatomic distances between the donor atoms of carnosine and ruthenium in complex 2B were also found to be comparatively shorter than those present in the complexes 2A. These results also suggest that tetrahedral complex 2B including its carboxylate and imidazole moieties in complexation is more compact and stable than complexes 2A and 2C. A simple method for the preparation of ruthenium(II)-carnosine complexes in aqueous solutions was demonstrated. The feasibility of carnosine-ruthenium complex synthesis and characterization may be very important from the drug design point of view. Further studies involving the detailed characterization of carnosine-ruthenium complex, in conjunction with computational studies are currently underway in our laboratory.
